# Wide-field fundus autofluorescence imaging in patients with hereditary retinal degeneration: a literature review

**DOI:** 10.1186/s40942-019-0173-z

**Published:** 2019-12-12

**Authors:** Akio Oishi, Manabu Miyata, Shogo Numa, Yuki Otsuka, Maho Oishi, Akitaka Tsujikawa

**Affiliations:** Department of Ophthalmology and Visual Sciences, Kyoto University Graduate School of Medicine, Shogoin Kawahara-cho 54, Sakyo-ku, Kyoto, Japan

**Keywords:** Retinal degeneration, Retinal dystrophy, Retinitis pigmentosa, Cone-rod dystrophy, Fundus autofluorescence, Wide angle, Optos, Optomap, Confocal scanning laser opthalmoscope

## Abstract

**Background:**

Inherited retinal degeneration (IRD) refers to a heterogenous group of progressive diseases that cause death of photoreceptor cells and subsequent vision loss. These diseases often affect the peripheral retina, objective evaluation of which has been difficult until recently. Fundus autofluorescence (FAF) is a non-invasive retinal imaging technique that depicts the distribution of intrinsic fluorophores in the retina. The primary source of retinal autofluorescence is lipofuscin, which is contained in the retinal pigment epithelium (RPE). Excessive accumulation of lipofuscin and a window defect attributable to loss of photoreceptor pigment result in increased FAF whereas loss of the RPE results in decreased FAF. These changes can be seen during the course of IRD.

**Mainbody:**

While conventional modalities are limited in their angle of view, recent technologic advances, known as wide-field and ultra-widefield FAF imaging, have enabled visualization of the far peripheral retina. Although clinical application of this technique in patients with IRD is still in its infancy, some studies have already indicated its usefulness. For example, an area with decreased FAF correlates well with a visual field defect in an eye with retinitis pigmentosa (RP) or cone-rod dystrophy. An abnormal FAF pattern may help in the diagnosis of IRD and associated diseases. In addition, female carriers of X-linked RP and female choroideremia show characteristic appearance. Conversely, absence of abnormal FAF despite severe retinal degeneration helps differentiation of cancer-associated retinopathy.

**Conclusion:**

This paper reviews the principles of FAF, wide-field imaging, and findings in specific diseases. Wide-field imaging, particularly wide-field FAF, will provide further information for the characteristics, prognosis, and pathogenesis of IRD.

## Background

### Inherited retinal degeneration

Inherited retinal degeneration (IRD) encompasses a heterogenous group of diseases that are characterized by retinal degeneration. Other terms, such as “inherited retinal dystrophy” and “inherited retinal disease” are often used. While the term “dystrophy” has been used to indicate progressive involvement of specific cells in the presence of a genetic cause and the term “degeneration” indicates progressive loss of function irrespective of whether a disorder is genetic or acquired [1], these terms are usually used without specific differentiation to describe IRD in clinical practice.

IRD can be inherited in an autosomal dominant, autosomal recessive, X-linked recessive, and occasionally digenic or mitochondrial manner. It can be caused by mutations in genes involved in any retinal characteristic, including development, phototransduction, visual cycle, ciliary trafficking, ion channels, lipid metabolism, mitochondrial function, and RNA splicing [2]. According to RetNet (https://sph.uth.edu/retnet/), more than 250 causative genes have been identified to date. Depending on the type of cell primarily affected, IRD can be categorized into rod-dominant, cone-dominant, generalized retinal degenerative, and vitreoretinal disorders [3]. While retinal prostheses [4] and gene therapy [5] have been successful in some patients, there is still no established treatment for the entire IRD population. Thus, IRD remains an important cause of blindness in developed countries [6].

IRD is called syndromic when multiple organs are affected. For example, retinal degeneration accompanied by sensorineural hearing loss occurs is called Usher syndrome, and retinal degeneration complicated by the combination of obesity, polydactyly, hypogonadism, and renal failure is called Bardet-Biedl syndrome [7]. The retinal phenotype is essentially identical in syndromic and non-syndromic IRD and cannot be differentiated by retinal examination alone.

## Main text

### Principles of fundus autofluorescence

Fundus autofluorescence (FAF) is a relatively new functional imaging technique for evaluation of the metabolism of photoreceptor cells and the retinal pigment epithelium (RPE). The examination uses a light source with a specific wavelength to excite intrinsic fluorescent material in the retina.

Autofluorescence phenomena had already been recognized when fundus fluorescein angiography first entered clinical use in the 1960s. The light emitted from the human retina by the same excitation light used in fluorescein angiography but without injection of fluorescein dye was known as “pseudofluorescence” [8]. While the fluorescence was partly explained by the overlap of wavelength between excitation light and emission filter, retinal autofluorescent materials also contributed to the phenomenon. After several investigations, lipofuscin that accumulates in the RPE was identified as the primary fluorophore that caused pseudofluorescence in the human eye [9–11].

Lipofuscin is derived mainly from components of the discs in the outer segment of the photoreceptor, particularly 11-cis-retinal, which is produced in the visual phototransduction cycle. The RPE continuously phagocytoses discs in the outer segments of photoreceptors. It is estimated that more than 3,000,000,000 discs are phagocytosed during a human lifetime [12]. Thus production, phagocytosis, and degradation of the discs are totally physiologic processes. However, the outer segments of photoreceptors that degenerate in response to oxidative or age-related stressors are less susceptible to degradation by lysosomal enzymes, and the metabolic products of phagocytosis accumulate as lipofuscin with aging [13, 14]. Lipofuscin is excited by light at wavelengths of 300–600 nm and emits light at 480–800 nm with a maximum at 600–640 nm.

Two types of imaging devices are commercially available for recording FAF, i.e., the fundus camera and the confocal scanning laser ophthalmoscope (cSLO). The fundus camera records all the reflected light whereas the cSLO contains a small aperture that blocks the scattered light from adjacent tissue and provides better resolution and contrast. The principal component of lipofuscin is N-retinyl-N-retinylidene ethanolamine (A2E), which is known to be toxic to the RPE and was originally considered to have a major role in the pathologic effect of lipofuscin on the retina [15, 16]. However, a more recent study showed that A2E is distributed mainly in the peripheral retina and that there is no correlation between the A2E level and the intensity of autofluorescence of lipofuscin [17]. More studies are needed to determine how accumulation of lipofuscin and increased FAF correlate with retinal pathology.

For historical reasons, FAF has been recorded mainly using light with a short wavelength. However, intrinsic fluorophores can also be excited at longer wavelengths, such as near infrared light at 787 nm. The main fluorophore in these infrared autofluorescence images is thought to be melanin in the RPE and choroidal tissue [18]. However, although infrared autofluorescence provides valuable information and the longer wavelength light causes less discomfort to patients than light of short wavelengths, the existing knowledge regarding FAF is based on short wavelength light, so interpretation of infrared autofluorescence images is presently not straightforward. Therefore, in this review, FAF refers to short-wavelength FAF unless otherwise specified.

### Interpretation of fundus autofluorescence

In principle, the intensity of the autofluorescence signals depends on the concentration of lipofuscin, and this has allowed researchers to evaluate the status of photoreceptors and the RPE cells. However, the signal intensity can be affected by a complex interaction between the distribution of fluorophores, structure of the retina, and pathologic changes, as discussed in the following sections.

#### Physiologic changes

Normal eyes emit a diffuse background FAF signal throughout the retina except at the fovea, optic disc, and retinal vessels. In normal eyes, the FAF signal emitted by the fovea is weaker because of absorption of light by the luteal pigment and the higher optical density of melanin in the central RPE [19]. The signal emitted in the juxtafoveal area is slightly stronger than that at the fovea but is still relatively weak in comparison with the background level. The optic disc has a dark appearance because of the absence of the RPE and lipofuscin. The retinal vessels also emit a weaker FAF signal as a result of absorption of excitation/emission light by blood (mainly red blood cells) in the vessel. Structures anterior to the retina, including the cornea, vitreous, and in particular the lens, which is a fluorescent organ in itself, may interfere with FAF images [12].

The intensity of FAF images is determined by multiple factors, including age, media opacity, pupil diameter, and light exposure, so interindividual or even intraindividual comparisons require calibration [20]. Therefore, identification of an abnormal finding basically relies on deviation from the normal distribution or from the signal of intensity of surrounding area. Abnormal FAF findings can be classified as increased or decreased.

#### Interpretation of increased FAF

Increased FAF occurs primarily because of an increased amount of or compositional change in the fluorophore. For example, excessive accumulation of lipofuscin is observed in Stargardt disease, pattern dystrophy, Best disease, and adult-onset vitelliform dystrophy [12, 21]. The hypotheses put forward to explain why FAF is increased in the diseased retina include accelerated phagocytosis of the outer segments of photoreceptors and initiation of a pathway for accelerated synthesis of lipofuscin in abnormal photoreceptor cells. Impairment of degradation process due to dysfunction of the RPE may result in the increased amount of lipofuscin. Photooxidative modification of lipofuscin in the RPE is presumed to be an example of compositional change in a fluorophore [22]. Fluorophores other than lipofuscin, such as optic disc drusen, can also emit a strong FAF signal.

A window defect, i.e., loss of the materials that block or absorb autofluorescence, is another cause of increased FAF. For example, depletion of luteal pigment in the macula is involved in development of increased FAF at the fovea in patients with macular telangiectasia type 2 [23]. This phenomenon is well known in macular telangiectasia; however, other diseases, such as Sjogren-Larsson syndrome, may show the same phenomenon [24]. Indeed, disruption of the outer retina and a consequent reduction in optical pigment density may cause increased FAF in any retinal diseases [25].

#### Interpretation of decreased FAF

Decreased autofluorescence can occur because of an absence or reduction of lipofuscin or the presence of a blocking material anterior to the RPE and photoreceptors. For example, loss of RPE cells and consequent loss of the fluorophores contained in those cells results in decreased FAF. This is common to most types of IRD and is also found in atrophic age-related macular degeneration and RPE tear. The reduced visual cycle in fundus albipunctatus or *RPE65*-associated leber congenital amaurosis may be associated with a diffuse decrease in FAF.

Autofluorescence may also be blocked by material lying anterior to the RPE/photoreceptors, including media opacities, intraretinal or subretinal fluid/lipid/hemorrhage, pigmentation, and fibrosis or scar tissue. For example, bony spicule pigmentation in RP causes decreased FAF.

### Ultra-widefield FAF

**Fig. 1:**
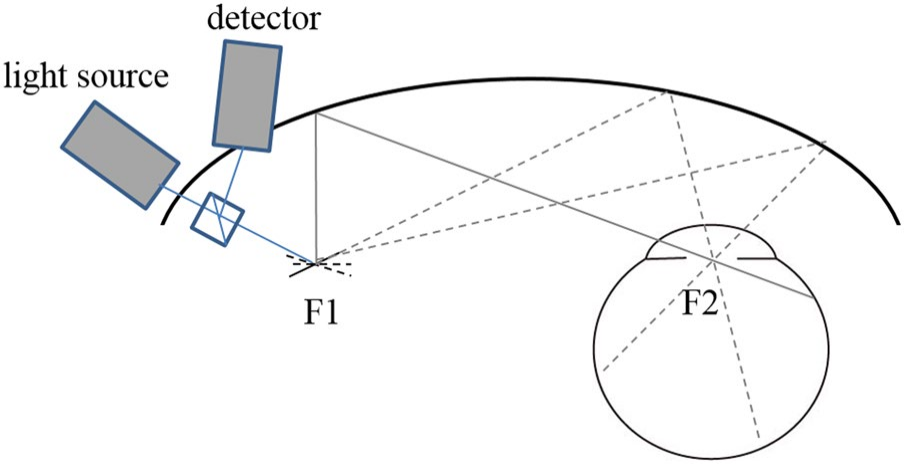
Principle of ultra-widefield imaging using the confocal scanning laser ophthalmoscope developed by Optos. An ellipse has two foci, and light passing through one focus and reflecting at the inner surface of the ellipse always passes through the other focus. Thus, light emitted from one of the foci (F1) always passes through the other focus (F2) where the center of the pupil is placed. The direction of light can be changed when a galvanometer mirror is placed at F1. Taking advantage of this property, ultra-widefield retinal imaging of even a small pupil became possible

FAF was originally recorded using a fundus camera or scanning laser ophthalmoscope that covered up to 55° of the retina and was limited in angle of view. Recently, an ultra-widefield cSLO was developed and commercialized by Optos PLC (Dunfermline, Scotland, UK). The Optos instrument uses an ellipsoid mirror that reflects the laser. An ellipse has two foci in principle, and light passing through one of the foci always passes through the other. Thus, setting a galvanometer mirror at one focus and the center of the pupil at the other focus allows scanning of up to 200° of the retina without dilation of the pupil (Fig. 1). Since the retinal pathology of IRD is not limited to the macula, wide-field FAF provides valuable and otherwise inaccessible information.

Attachment of special lenses to a conventional cSLO also enables FAF to be recorded with a large field of view. For example, the Staurenghi lens can visualize 150° of the retina and the Heidelberg ultra-widefield lens can depict up to 105° with less artifact and more uniform contrast than the Optos [26]. However, while these modalities are also useful, the Staurenghi lens is a contact lens and the Heidelberg ultra-widefield lens has a slightly smaller angle of view than the Optos. Therefore, in this review, ultra-widefield FAF refers to imaging by the Optos device unless otherwise specified.

### Difference between conventional and ultra-widefield FAF

**Fig. 2:**
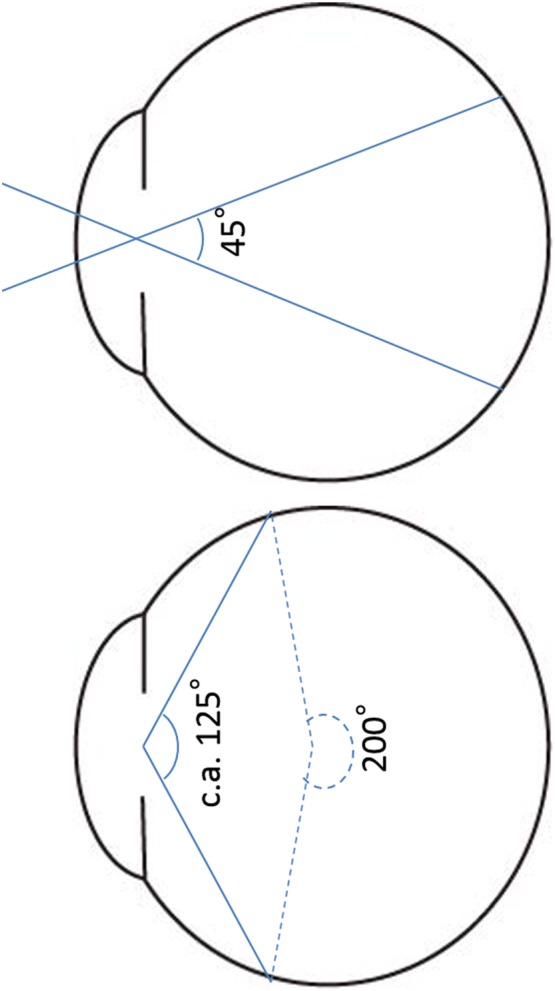
Comparison of conventional measurement of the angle of view and the angle used by the Optos system. Whereas the angle of view has traditionally referred to the angle at which light enters the eye, the Optos measures the angle from the center of the eye. The 200° from the center of the eye corresponds to approximately 125° measured in the conventional manner

The primary difference between conventional and ultra-widefield FAF is the angle of view. The angle of view of a fundus camera and that of a conventional cSLO is up to 60°, whereas the Optos device can visualize up to 200°. When considering the angle of view, the reader should bear in mind that these numbers are not measured using the same scale. Whereas a conventional fundus camera measures the angle of light that enters the eye, the Optos measures the angle from the center of the eye. For example, 200° from the center of the eye corresponds to a conventional measurement of approximately 125° (Fig. 2).

Another difference is the wavelength of excitation light and the barrier filter used, which are specific for each device. The standard cSLO system (the Heidelberg retinal angiograph) uses 488 nm (blue) light for excitation and > 500 nm for the emission filter, whereas the Optos system uses 532 nm (green) light for excitation and 570–780 nm for the emission filter [27]. The Optos is also equipped with 488 nm (blue) light but this is for fluorescein angiography and cannot be used to acquire FAF images. Therefore, the images may contain inherent differences, especially when evaluating the fovea [28]. Although previous studies indicate that these differences have little impact when evaluating the peripheral area in an eye with IRD, they should be kept in mind.

**Fig. 3:**
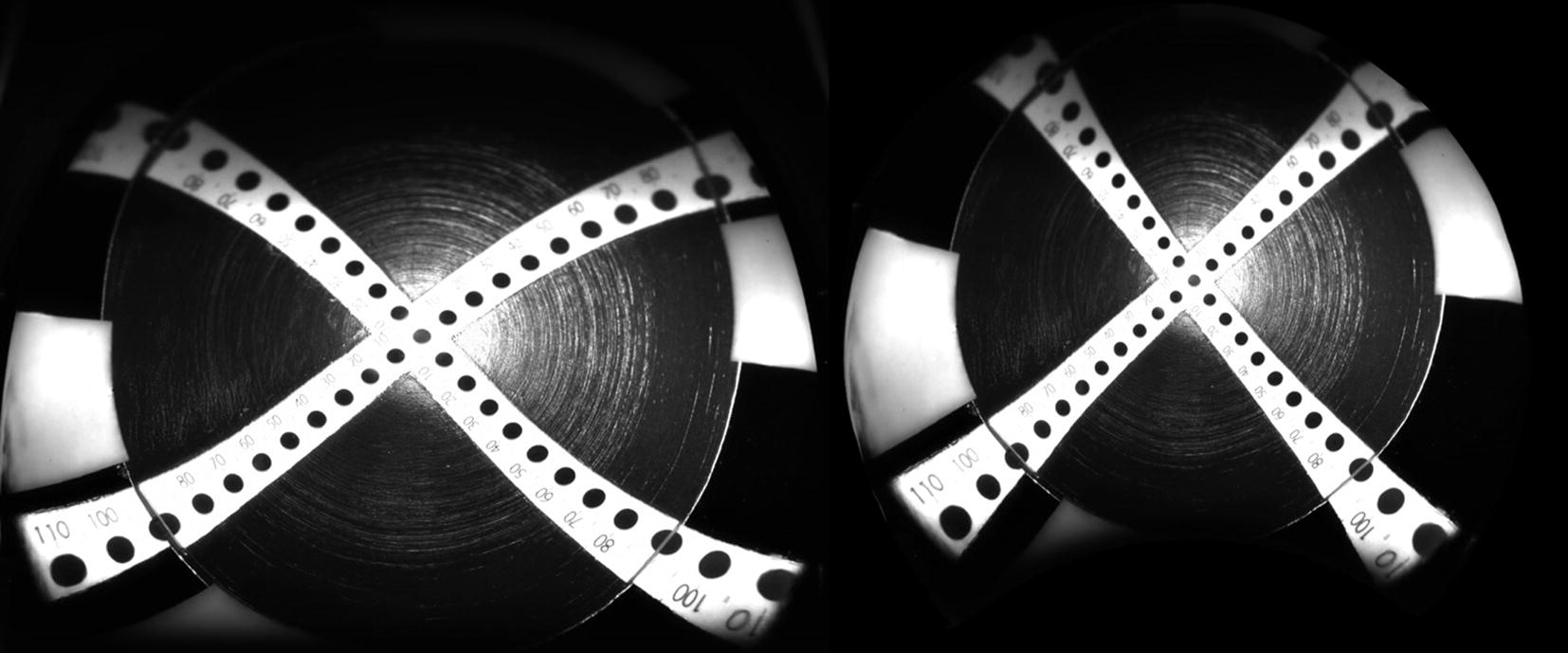
Example of image distortion in response to transformation of the sphere into a plain image. We took a retinal image using a model eye with a vertical scale inside. Note that peripheral portion is magnified, and the vertical scale is distorted. (left) The Optos algorithm compensates for this distortion. (Right) Recent models have an additional algorithm to measure the theoretically correct length and area on the image

Distortion is another important issue in ultra-widefield images. In principle, it is impossible to transform a three-dimensional sphere into a completely accurate two-dimensional plain image [29], and distortion of the angle, length, and/or area is inevitable. When handling the posterior pole, the depth of the spherical cap is limited and can be approximated to the plain image. However, this is not the case with ultra-widefield images. The most peripheral area of the retina is considerably enlarged in the display [30]. Furthermore, the overall image is stretched horizontally, and the image contrast is limited in the superior and inferior quadrants, probably due to asymmetric optical properties of the ellipsoid mirror [30] (Fig. 3a). To address this issue, Optos first provided a program to display the image with the correct angle. If the upper and lower part of the image is displayed in a convex manner, the image is adjusted for the angle (Fig. 3b). Later, Optos, in collaboration with the team led by Professor Sadda, developed a program to transform plain images into stereographic projection images and obtain theoretically precise measurements of length and area [31, 32]. This program is now commercially available.

### Clinical application of ultra-widefield FAF in inherited retinal degeneration

#### Retinitis pigmentosa

Retinitis pigmentosa (RP) is a clinical diagnosis and includes a genetically heterogenous group of patients with rod-dominant retinal degeneration and a classic triad of bony spicule pigmentation, vessel attenuation, and a waxy pallid optic disc. These patients initially suffer from night blindness, which is followed by constriction of the visual field, and finally impaired visual acuity. Most syndromic variants of IRD, such as Usher syndrome and Bardet-Biedl syndrome, have the retinal phenotype of RP. Thus far, at least 120 genes are reported to cause syndromic or non-syndromic RP (RetNet, https://sph.uth.edu/retnet/). The prevalence of causative genes may differ according to ethnicity or local population [33].

An increased FAF ring in the macula in patients with RP was reported in the 2000s [34, 35]. This ring is considered to represent the transition between the degenerating retina and the relatively normal retina. Morphologic spectral-domain optical coherence tomography studies showed preservation of the ellipsoid zone (previously known as the “inner segment/outer segment junction”) inside the ring [36, 37]. Similar findings have been reported in Leber congenital amaurosis [38, 39]. A patient’s visual function can be roughly estimated based on a simple classification of no ring, evident ring, and increased FAF in the fovea [40]. The ring decreases in size along with the functional decline during the course of the disease [41]. Double or triple concentric rings are sometimes seen in patients with mutations in a specific gene [42]. Most studies of the increased FAF have used blue FAF; however, the ring can also be seen using the green FAF of the Optos device [43].

**Fig. 4:**
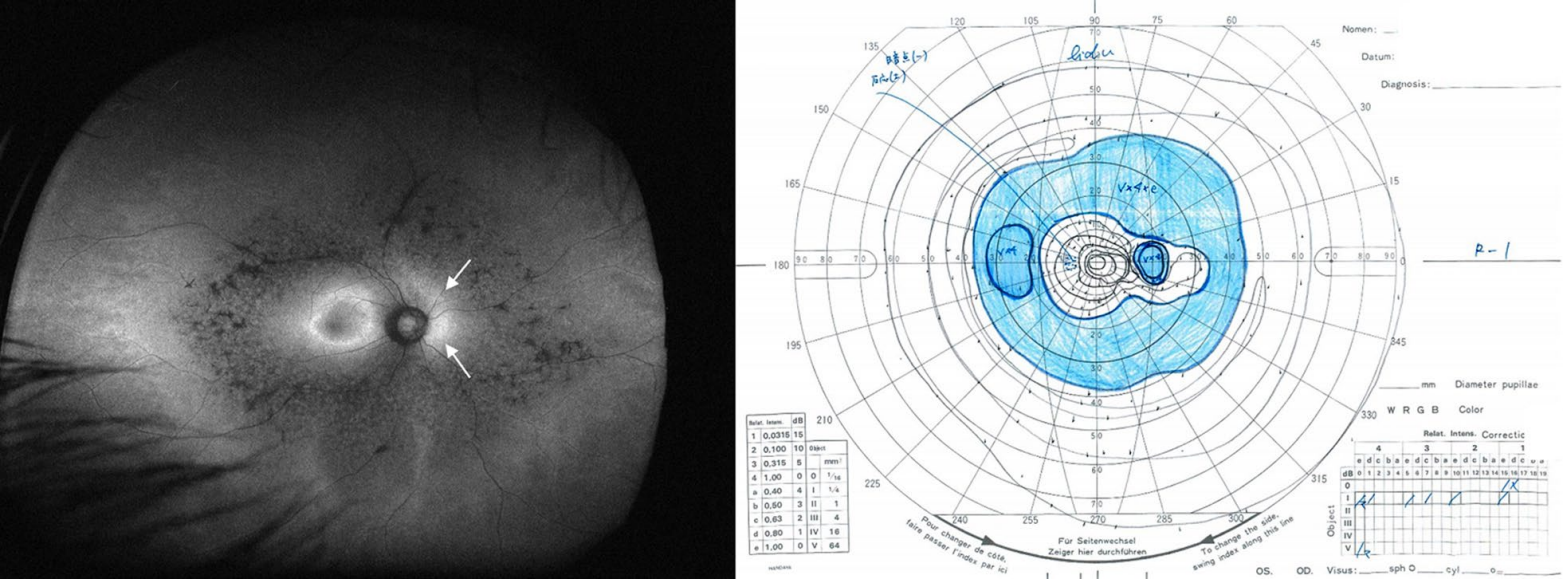
An ultra-widefield fundus autofluorescence (FAF) image and Goldmann perimetry of an eye with retinitis pigmentosa. An increased ring of FAF is observed in the macula. In addition, the retina nasal to the optic disc shows slightly stronger FAF despite decreased FAF in the surrounding area. (arrows) This phenomenon is known as nasal sparing and is a characteristic finding in retinitis pigmentosa. The visual field results correspond well with the FAF findings

**Fig. 5:**
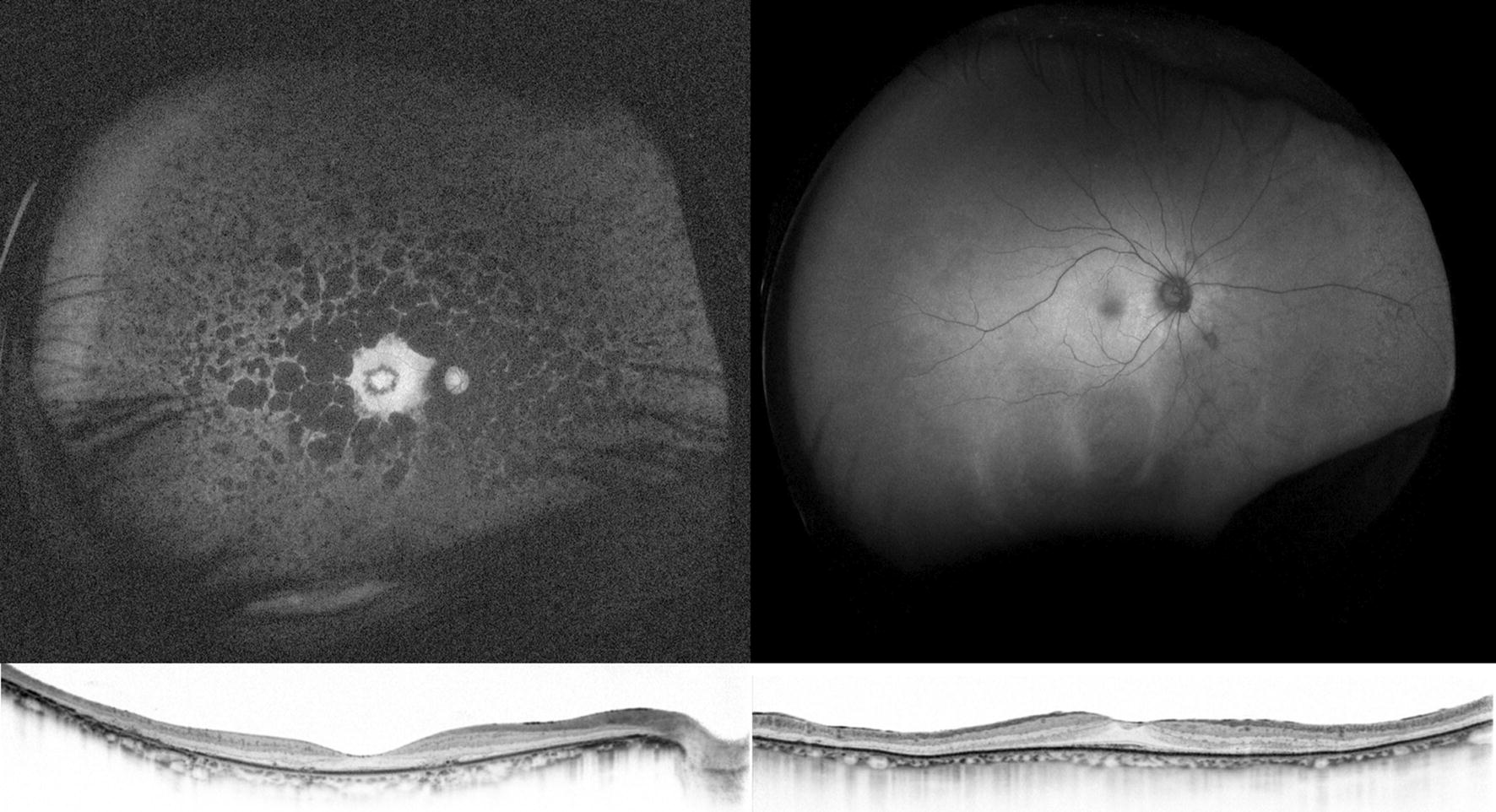
Optical coherence tomography and ultra-widefield fundus autofluorescence (FAF) in a 59-year-old patient with retinitis pigmentosa (RP; left) and an 82-year-old patient with subacute vision loss and night blindness (right). Optical coherence tomography revealed thinning of the paracentral outer nuclear layer and loss of the ellipsoid zone in these two patients. However, ultra-widefield FAF shows minimal changes in the patient on the right compared to the patient on the left. In RP, development of a granular and patchy area of decreased FAF depends on patient age and duration of the disease [43]. Thus, it is very unlikely that a patient with RP in the ninth decade of life would show almost normal FAF. The patient was screened for a systemic tumor, and lung cancer was detected. The patient was finally diagnosed with cancer-associated retinopathy

Ultra-widefield FAF provides additional information on the state of the peripheral retina. Since rod photoreceptors are distributed widely throughout the retina, evaluation of the peripheral retina is particularly important in this rod-dominant disease. A granular and patchy area of decreased FAF can be seen in patients with RP. The distribution pattern of the decreased FAF is generally symmetric in both eyes and similar in family members [44]. Interestingly, the decreased FAF lesion tends to spare the retina nasal to the optic disc. This is known as “nasal sparing” and is considered to result from distribution of cone photoreceptors [45]. The area of decreased FAF is associated with a visual field defect [43] and the shape of this decreased area corresponds spatially with the pattern of visual field defect [46] (Fig. 4). Furthermore, the wide-field FAF provides insight into the duration of the disease. The number of patchy areas of decreased FAF increases with age and disease duration [43]. This is particularly useful when the subjective symptoms are unclear and differentiation of other diseases is required. In such cases, asymptomatic but chronic disease can be assumed if a considerable patchy area of decreased FAF is confirmed. If there is no patchy area of decreased FAF despite evident visual field loss, more rapidly progressive diseases, such as an autoimmune or cancer-associated retinopathy, must be considered. An example is shown in Fig. 5.

### Choroideremia

**Fig. 6:**
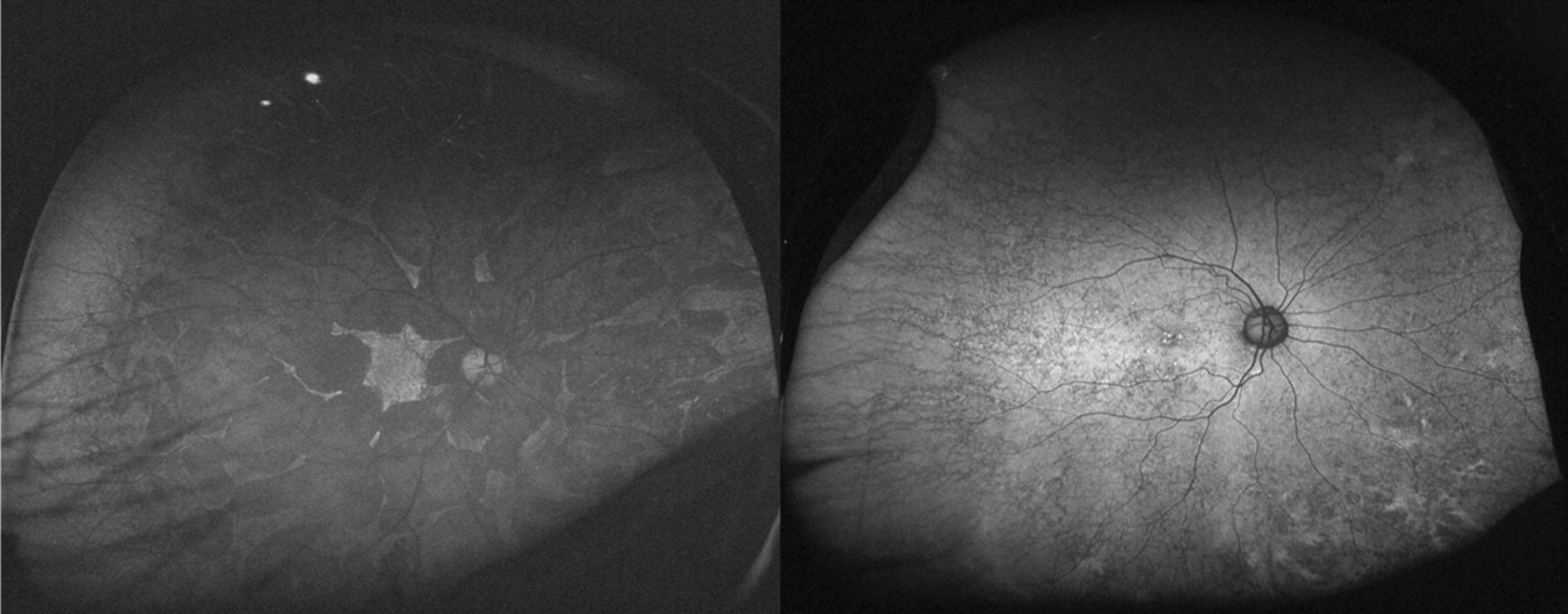
Ultra-widefield fundus autofluorescence (FAF) images of a patient with choroideremia (left) and his mother (right). Patients with choroideremia show patchy or granular areas of decreased FAF throughout the retina. Large choroidal vessels are sometimes visible in the decreased FAF area. Interestingly, an asymptomatic female carrier may show an abnormal reticular or mottled pattern of FAF. This finding may help in the diagnosis

Choroideremia is a progressive X-linked IRD that affects the RPE, choroid, and retina. Loss of night vision begins in the first decade of life in affected individuals and progresses to a gradual loss of peripheral vision and then to legal blindness by the fifth decade [47]. The bony spicule pigmentation, vessel attenuation, and waxy pallid optic disc is absent or less evident in choroideremia, which helps in differentiation from RP. Causative gene *CHM* was identified in 1990 [48]. A FAF image in a patient with choroideremia contains patchy irregular patterns extending from the posterior pole to the periphery (Fig. 6a). The area of residual FAF in an eye with choroideremia can be measured reproducibly [49]. The residual area of FAF in choroideremia decreases its size at a rate of 7.7% per year. Moreover, the residual area measured on the FAF image can be used to assess the effect of retinal gene therapy [50].

Female carriers, although asymptomatic and able to maintain good visual function lifelong, usually show changes at the fundus, including patchy depigmentation of the RPE and coarse pigmentary granularity in the periphery [51]. The abnormality in the peripheral retina can be appreciated using ultra-widefield FAF [52] (Fig. 6b). Screening of the peripheral retina is also useful in family members of X-linked RP. Female carriers with heterozygous mutations of the X-linked RP gene, particularly *RPGR*, also show an abnormal radial pattern of increased FAF. While this finding can be visualized by standard FAF [53], it is more easily recognizable by ultra-widefield FAF [54].

### Cone/cone-rod dystrophy

Cone dystrophy (CD) and cone-rod dystrophy (CRD) comprise a group of cone-dominant retinal dystrophies for which least 34 causative genes have been reported to date (RetNet, https://sph.uth.edu/retnet/) [3]. Patients are diagnosed with CD when rod function is retained and CRD when both cone and rod function are impaired. However, the distinction between CD and CRD is now less clear than previously thought; patients initially diagnosed with CD can also develop rod dysfunction when the condition is advanced [55]. There is also considerable overlap of both the phenotype and the causative genes between CD/CRD and RP [56].

In the early stages of the disease, patients with CD/CRD show impairment in visual acuity and/or color vision. Photophobia is also a common symptom. The fovea may show atrophic changes in affected individuals but may look normal in some cases. Increased FAF at the fovea is sometimes observed even in eyes with normal funduscopy, which provides a diagnostic clue [57]. A ring of increased FAF comparable with that in RP is also seen in CD/CRD. However, unlike RP, the retina is impaired inside the ring and the ring increases in size as the disease progresses [58].

**Fig. 7:**
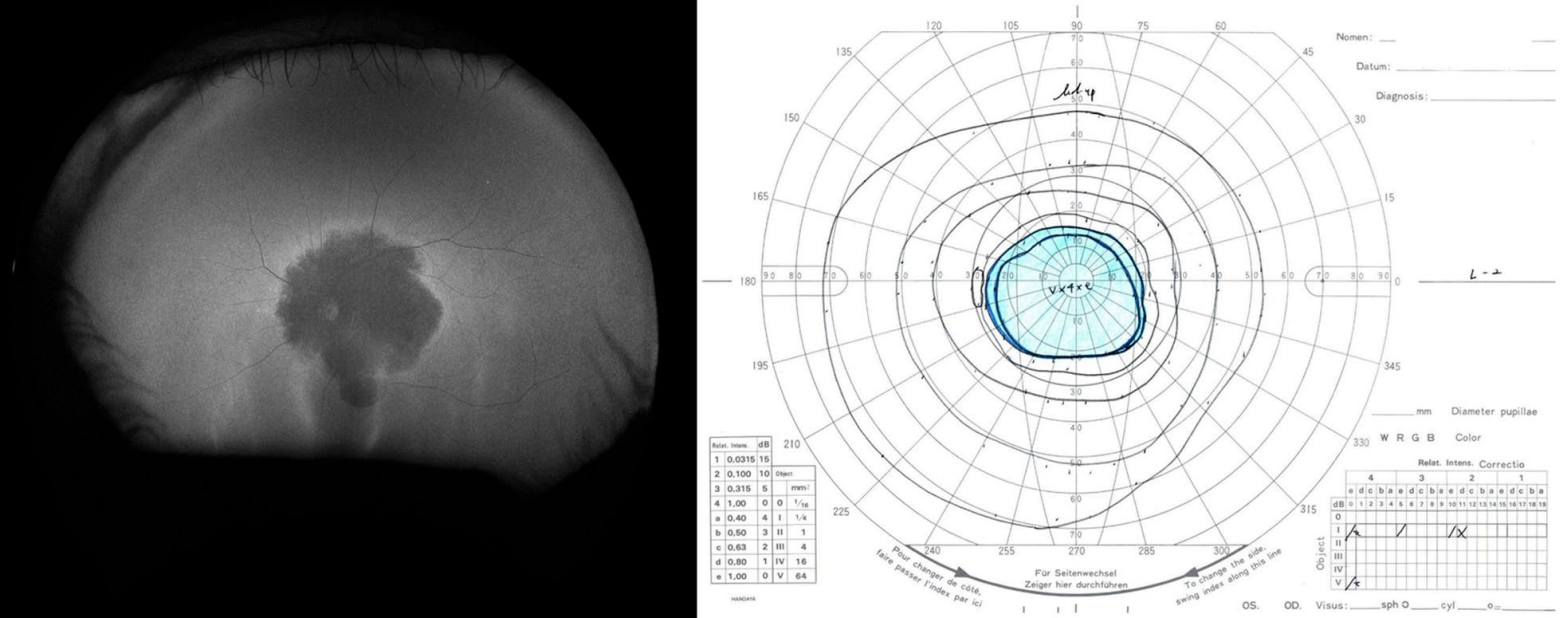
An ultra-widefield fundus autofluorescence (FAF) image and Goldmann perimetry of a patient with cone-rod dystrophy. The macula is severely affected and shows decreased FAF. Perimetry confirmed a central scotoma corresponding to this area

Although impairment of central vision is the primary symptom of both CD and CRD, these diseases are essentially pan-retinal dystrophies and may affect the peripheral retina. A previous study that used wide-field FAF showed that the extent of abnormal FAF correlates with visual function in patients with CD or CRD [59]. The area of abnormal FAF was associated with the size of the scotoma measured by Goldmann kinetic perimetry (I/4e white test light; Fig. 7). This study also suggested that the location and size of the scotoma correspond spatially to the area of abnormal FAF, as in patients with RP. The area of abnormal FAF also correlated well with the amplitude of the full-field electroretinogram under all conditions. The correlation was relatively strong for rod and combined responses and moderate for cone and flicker responses. Thus, wide-field FAF, which can evaluate the entire retina, is clinically useful for evaluating visual function in patients with CD and CRD.

### Stargardt disease

Stargardt disease was first described in 1909 [60]. This disease has an autosomal recessive inheritance trait and is characterized by foveal atrophy and yellowish flecks around an atrophic area. Mutations in the *ABCA4* gene have been identified as a cause of the disease. On rare occasions, mutations in other genes, such as *ELOVL4* [61] and *PROM1* [62], may be expressed as autosomal dominant Stargardt disease-like macular dystrophy. Multifocal pattern dystrophy caused by mutations in *PRPH2* may also mimic Stargardt disease [63].

The characteristic fundus flecks in Stargardt disease show increased FAF, which indicates accumulation of lipofuscin. The atrophic macular area shows decreased FAF and is associated with electroretinographic responses [64]. The characteristic flecks develop and disappear during the course of the disease. They extend centrifugally from the fovea, and the flecks with increased FAF over time start to show decreased FAF [65]. Typically, Stargardt disease affects only the macular area and the lesion can be observed with conventional FAF [66]. The peripapillary sparing characteristic of the disease can also be evaluated using conventional FAF [67].

**Fig. 8:**
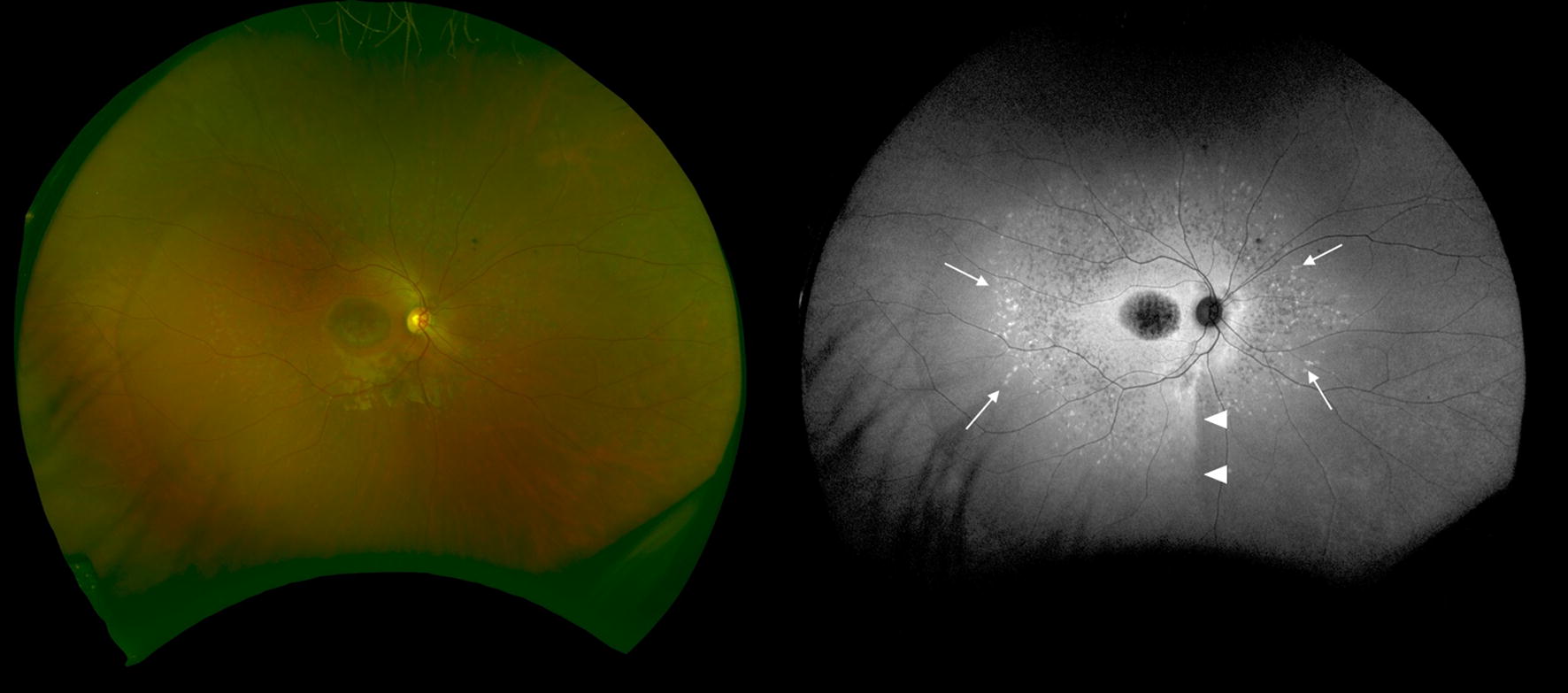
An ultra-widefield fundus photograph and a fundus autofluorescence (FAF) image of a patient with Stargardt disease. An area of foveal atrophy shows decreased FAF. No characteristic flecks are evident on the photograph but easily recognizable areas of increased FAF were detected (arrows). A demarcation line is often observed in this disease as well as in retinitis pigmentosa and is considered to represent a closed optic fissure [71] (arrowhead)

Occasionally, the atrophic lesion at the fovea expands, and the flecks become less evident in the macular area. In such situations, wide-field FAF may provide a diagnostic clue or help with monitoring (Fig. 8). A previous study showed that abnormal FAF in the periphery corresponds to the visual field loss and decreased amplitude on a full-field electroretinogram [68]. Another small case series reported the presence of a peripheral pigmented retinal lesion that was associated with poor visual function [69]. Thus, ultra-widefield FAF also provides previously unavailable information in macula-dominant disease.

## Conclusions

During the last decade, ultra-widefield FAF imaging, particularly using the Optos cSLO, has advanced our knowledge of retinal pathology. Application of this technology to IRD has barely started. Future challenges include determining the relationship between ultra-widefield FAF patterns and causative genes [70]. Identification of specific findings related to a good or poor prognosis would also be helpful. Although current information relies mostly on green FAF, other wavelengths, such as near infrared, may provide further information. These issues should be addressed in future research.

## Data Availability

Not applicable.

## Abbreviations

CD: cone dystrophy
CRD: cone rod dystrophy
cSLO: confocal scanning laser ophthalmoscope
FAF: fundus autofluorescence
IRD: inherited retinal degeneration
RP: retinitis pigmentosa
RPE: retinal pigment epithelium
